# Characterizing and Identifying the Prevalence of Web-Based Misinformation Relating to Medication for Opioid Use Disorder: Machine Learning Approach

**DOI:** 10.2196/30753

**Published:** 2021-12-22

**Authors:** Mai ElSherief, Steven A Sumner, Christopher M Jones, Royal K Law, Akadia Kacha-Ochana, Lyna Shieber, LeShaundra Cordier, Kelly Holton, Munmun De Choudhury

**Affiliations:** 1 University of California, San Diego San Diego, CA United States; 2 Office of Strategy and Innovation National Center for Injury Prevention and Control Centers for Disease Control and Prevention Atlanta, GA United States; 3 National Center for Injury Prevention and Control Centers for Disease Control and Prevention Atlanta, GA United States; 4 Division of Injury Prevention National Center for Injury Prevention and Control Centers for Disease Control and Prevention Atlanta, GA United States; 5 Brunet-García Atlanta, GA United States; 6 School of Interactive Computing Georgia Institute of Technology Atlanta, GA United States

**Keywords:** opioid use disorder, substance use, addiction treatment, misinformation, social media, machine learning, natural language processing

## Abstract

**Background:**

Expanding access to and use of medication for opioid use disorder (MOUD) is a key component of overdose prevention. An important barrier to the uptake of MOUD is exposure to inaccurate and potentially harmful health misinformation on social media or web-based forums where individuals commonly seek information. There is a significant need to devise computational techniques to describe the prevalence of web-based health misinformation related to MOUD to facilitate mitigation efforts.

**Objective:**

By adopting a multidisciplinary, mixed methods strategy, this paper aims to present machine learning and natural language analysis approaches to identify the characteristics and prevalence of web-based misinformation related to MOUD to inform future prevention, treatment, and response efforts.

**Methods:**

The team harnessed public social media posts and comments in the English language from Twitter (6,365,245 posts), YouTube (99,386 posts), Reddit (13,483,419 posts), and Drugs-Forum (5549 posts). Leveraging public health expert annotations on a sample of 2400 of these social media posts that were found to be semantically most similar to a variety of prevailing opioid use disorder–related myths based on representational learning, the team developed a supervised machine learning classifier. This classifier identified whether a post’s language promoted one of the leading myths challenging addiction treatment: that the use of agonist therapy for MOUD is simply replacing one drug with another. Platform-level prevalence was calculated thereafter by machine labeling all unannotated posts with the classifier and noting the proportion of myth-indicative posts over all posts.

**Results:**

Our results demonstrate promise in identifying social media postings that center on treatment myths about opioid use disorder with an accuracy of 91% and an area under the curve of 0.9, including how these discussions vary across platforms in terms of prevalence and linguistic characteristics, with the lowest prevalence on web-based health communities such as Reddit and Drugs-Forum and the highest on Twitter. Specifically, the prevalence of the stated MOUD myth ranged from 0.4% on web-based health communities to 0.9% on Twitter.

**Conclusions:**

This work provides one of the first large-scale assessments of a key MOUD-related myth across multiple social media platforms and highlights the feasibility and importance of ongoing assessment of health misinformation related to addiction treatment.

## Introduction

### Background

In the United States, opioid overdose continues to be a leading cause of death [1]. The Centers for Disease Control and Prevention estimates that the total economic burden of prescription opioid misuse in the country alone is US $78.5 billion a year, including the costs of health care, lost productivity, treatment, and criminal justice involvement [2]. Alarmingly, opioid overdoses increased by 30% from July 2016 to September 2017 in 52 areas in 45 US states [3]. Consequently, in 2017, the Department of Health and Human Services declared it as a public health emergency [4]. Central to addressing the opioid crisis is expanding access to medication treatment for opioid use disorder (MOUD) [5]. MOUD increases treatment retention and reduces opioid use, risk behaviors that transmit blood-borne pathogens, and overdose mortality [6]. However, despite its well-documented effectiveness, studies have found that MOUD is underused due in part to stigma and misperceptions about treatment [7].

In recent years, many individuals have been seeking both conventional and nonconventional ways to recover from substance use, including using web-based resources [8]. For these conditions, as well as opioid use disorder (OUD), research has shown that individuals turn to the web for promoting and discovering recovery strategies, for example, appropriating the Forum77 forum for prescription drug use recovery [9] and participating in 12-step programs such as Narcotics Anonymous [10,11]. Social support is another motivation behind individuals with substance use disorders turning to social media; Rubya and Yarosh [12] examined peer support for substance use disorder recovery meetings through video chat, discovering that video chat support groups not only provide immediacy and convenience in meeting needs but can also be places of obtaining emotional and informational support. More recently, researchers have examined patterns of anonymity in web-based recovery communities [13]. Specific to OUD, previous studies have investigated the different types of web-based discourse associated with opioid use, including personal use, whether it is associated with legitimate use or abuse of opioids [14], or whether it involves the promotion of clinically unverified treatments [15]. Abuse discourse on social media platforms has been further broken down into stand-alone use and co-use of opioids with other opioids, illicit drugs, and alcohol [16]. In addition, a prior study analyzed the web-based discourse surrounding the perception of opioids [17]. The perception of opioids included commentary on the opioid crisis, opioids in general, and interaction with news surrounding the opioid crisis or medical use of opioids [17]. Researchers in the past have also harnessed social media data as unobtrusive sensors to identify individuals who might benefit from or be receptive to treatment and recovery interventions [18]. Others have computationally examined and compared web-based discussion communities to discover the intent to contribute to web-based mental health communities [19]. In general, social media platforms have been found to allow increased self-disclosure for users to discuss otherwise sensitive and stigmatizing topics such as OUD [20]. Apart from self-disclosure, social media data provide unique opportunities for understanding the users’ sentiments and opinions [21], which may be insightful from the perspective of addiction treatment.

Despite the positive benefits of social media, existing attempts of individuals with OUD are often challenged because of the pervasiveness of inaccurate and potentially harmful health misinformation on social media platforms [15]. Health misinformation is defined as a health-related claim of a fact that is currently false because of a lack of scientific evidence [22]. In general, misinformation is usually attributed to misconceptions and is not intended to cause harm. Disinformation is false information that is created deliberately to cause harm, with motivations that are often social, political, or financial. Although misinformation and disinformation are inherently false, malinformation is usually based on real information that is taken completely out of or without context to inflict harm [23]. Fake news is defined as fabricated information that mimics news media content in form but not in organizational process or intent [24,25]. Molina et al [24] have outlined key indicators of fake news such as content that is not fact-checked, is emotionally charged, is written in narrative style, has unverified sources, or comes from an unknown source. In this study, we focused on the language of false claims surrounding MOUDs regardless of intent; therefore, it might be the case that we captured a few instances of disinformation, possibly on web-based platforms that lack constant domain-specific moderation. Thus, we use the term *health misinformation* as we assume that the spread of these claims is not intentional.

From the discourse on infectious disease outbreaks and global epidemics to alternative therapies to tackle behavioral health problems, web-based misinformation can have adverse effects on public health, including negatively influencing people’s health literacy, attitudes, beliefs, and health-related decision-making [22]. For example, antivaccine-promoting social media posts legitimize debate about vaccine safety, contribute to reductions in vaccination rates, and increase vaccine-preventable diseases such as measles [26]. In the context of public health crises, social media rumors circulating during the Ebola outbreak in 2014 were found to create hostility toward health workers, which posed challenges in controlling the epidemic [27]. Most recently, the novel COVID-19 pandemic has come to be defined by a tsunami of persistent misinformation to the public on everything from the utility of masks and the effectiveness of social distancing to even the promise of vaccines, together contributing to an increased COVID-19 pandemic burden [28]. At-risk populations are known to be particularly vulnerable to misinformation [22,29] because of a lack of reliable information outside of formal clinical or rehabilitation contexts [30,31]. In fact, studies show that because of exposure to such misinformation, people worry that they will be ostracized by their community if their substance use is revealed to others, thus delaying treatment [32].

Given the limited uptake of MOUD, the potential contribution of health misinformation to this public health problem, and the fact that information about barriers to MOUD is challenging to ascertain from other data sources, exploring digital health-seeking behavior through *passive sensing* of misinformation related to MOUD provides an important avenue for addressing this problem. Thus, *infodemiology*, which refers to the science of studying the distribution and determinants of information and user-generated content in an electronic medium such as the web in general and social media in particular [33], has the opportunity to shape MOUD-related health promotion strategies and policies. Given the potential impact of misinformation in the midst of the ongoing overdose crisis, there is a critical need to better understand misinformation-related social media posts on OUD treatment. In fact, in recent years, approaches in infodemiology have been noted to be important in mitigating public health problems stemming from *infodemics* [34,35], a portmanteau of *information* and *epidemic* that typically refers to a rapid and far-reaching spread of both accurate and inaccurate information about a disease.

### Objective

In this study, we focus on one particular myth (and its language variants) related to MOUD: *agonist therapy or medication-assisted treatment (MAT) is simply replacing one drug with another*. For example, someone might express this myth by saying “You are not really in recovery if you are on Suboxone.” This myth is believed to be one of the major reasons cited for individual hesitancy to initiate MOUD; it has been discussed extensively in clinical literature [29,36,37] and has been discredited by evidence that MOUDs facilitate recovery and that multiple other chronic health conditions such as diabetes and asthma necessitate reliance on daily medication to maintain health.

By adopting a multidisciplinary, mixed methods strategy, this paper aims to present the first work that investigates the characteristics and prevalence of web-based misinformation related to MOUD across 3 types of web-based social platforms to inform future prevention, treatment, and response efforts. Our contributions include a set of machine learning (ML) models that classify whether a post revolves around conversations surrounding a specific MOUD as replacing one drug with another or explorations of lexical variations characterizing web-based conversations relating to this myth.

## Methods

### Data Set Curation

We first identified and curated a set of clinically grounded and publicly prevalent myths that surround OUD treatment and developed a lexicon of opioid-related keywords associated with different aspects of OUD. We captured different types of opioids, such as natural opiates, semisynthetic opioids, and synthetic opioids, and included opioids that were over-the-counter, prescription based, or illicit. For each generic name, we also included trade and combination product names in consultation with the substance use literature and the public health coauthors. This resulted in a total of 152 keywords curated in the lexicon. We then curated a diverse data set from Twitter, YouTube, and the web-based health communities Reddit and Drugs-Forum. These platforms were selected as (1) they are adopted pervasively by Americans and (2) there are well-established means and infrastructures for collecting meaningful data sets by leveraging app programming interfaces to query them and access public posts on these platforms. According to the Pew Research Center, in 2021, 18% of US adults use Reddit, 23% use Twitter, and 81% use YouTube [38]. In addition, these platforms have been mined in prior substance abuse literature for abuse monitoring and digital epidemiology purposes [39-41]. For all the platforms we investigated, we focused on public posts and messages created between January 1, 2018, and December 31, 2019.

Our data set collection methodology for Twitter comprised querying for all tweets that included 1 of the words in our lexicon. This process yielded a total of 6,365,245 tweets. For YouTube, owing to limitations in the number of comments that can be accessed, we restricted the 152 keywords to 11 OUD treatment keywords such as *buprenorphine* and *naltrexone*. We used the YouTube app programming interface to identify 552 YouTube videos that contained 1 of the 11 keywords in the title and then collected all of the associated comments (99,386 comments). We relied on expert domain knowledge to identify subforums pertinent to OUD for Reddit and Drugs-Forum and used the full set of 152 keywords for these sites. For Reddit, we used data from 22 opioid-specific subreddits: *r/Carfentanil*, *r/opiates*, *r/fentanyl*, *r/opiatesmemorial*, *r/modquittingkratom*, *r/Methadone*, *r/suboxone*, *r/kratom*, *r/heroin*, *r/quittingkratom*, *r/Tianeptine*, *r/loperamide*, *r/naltrexone*, *r/oxycodone*, *r/OpiatesRecovery*, *r/Opiatewithdrawal*, *r/lean*, *r/heroinaddiction*, *r/HeroinHeroines*, *r/OpiateChurch*, *r/suboxone*, and *r/OurOverUsedVeins*. This resulted in a total of 1,189,590 posts and 12,293,829 comments. In addition, we collected all 5549 messages posted under the *Opiates and Opioids* subforums on Drugs-Forum [42]. Throughout the paper, we have combined Reddit and Drugs-Forum content under the category of web-based health communities, as both have similar structure, format, and affordances.

### ML Approach Using Expert Involvement

Web-based discourse surrounding OUD is semantically rich; that is, there are different words and combinations of words that people use to convey meaning. Previous literature has quantitatively and qualitatively investigated various categories of language pertaining to OUD, including OUD use (own use, use by others, abuse, legitimate use, and co-use), OUD perception (commentary on opioid crisis or opioids in general), and OUD advertisements [14,16,17]. In light of such linguistic richness and prior investigations, we adopted an ML and natural language analysis methodology to identify posts relevant to the myth under investigation in the huge search space.

We first leveraged representation learning techniques, which are a set of techniques that allow a system to automatically discover the representations needed for feature detection or classification from raw data [43] to construct document-level embeddings (consisting of 4096 dimensions) of the myth statement noted earlier. For this, we used a bidirectional long short-term memory (LSTM) sentence encoder model universally trained on a natural language inference task [44]. LSTM was a suitable choice here as it allowed us to learn long-term dependencies among words in sentence structures. We then used this model to encode all the collected posts. Following this step, we obtained the k-nearest neighbor (KNN), where k=200, for semantically most similar posts per platform for the MOUD-related seed myth under investigation. Second, using a mixed methods approach, our models then harnessed qualitative content analysis in the form of public health expert annotations to label a total of 800 posts (200 KNNs per platform) and annotate whether each post was relevant to the myth (ie, whether the post discussed MOUD and described MOUD as using one drug to replace another). Hence, we modeled this problem as a binary classification task where the positive class denoted a post discussing the aforementioned piece of misinformation and the negative class represented any post that was not relevant to the myth. Each myth KNN post was annotated by the same expert public health annotator to provide consistent annotations within the linguistic domain of a given myth.

Leveraging these annotations as training data, we finally built and evaluated a series of supervised ML models, ranging from logistic regression (LR) and support vector machines to feedforward neural networks and LSTM networks. Our feature set included lexical features such as n-grams (n=1*,* 2*,* 3), term frequency–inverse document frequency (TF–IDF) weights, and representation learning features, including sentence-based embeddings (semantic) and transformer-based embeddings, such as bidirectional encoder representations from transformers [45] and bidirectional encoder representations from transformers for biomedical text mining [46]. We used all annotations belonging to our myth and considered all the samples from other myths as negative training samples. On the basis of this process, we obtained 171 positive samples and 2229 negative samples. Owing to this large imbalance, we leveraged an oversampling technique from the rare class, called the synthetic minority oversampling technique [47]. We then split the data set into training and test samples with an 80% to 20% split, respectively. We leveraged 2 techniques for cross-validation: k-fold cross-validation (for LR and support vector machine models) and an independent validation sample to tune a model’s hyperparameters (for the LSTM model).

## Results

**Figure 1:**
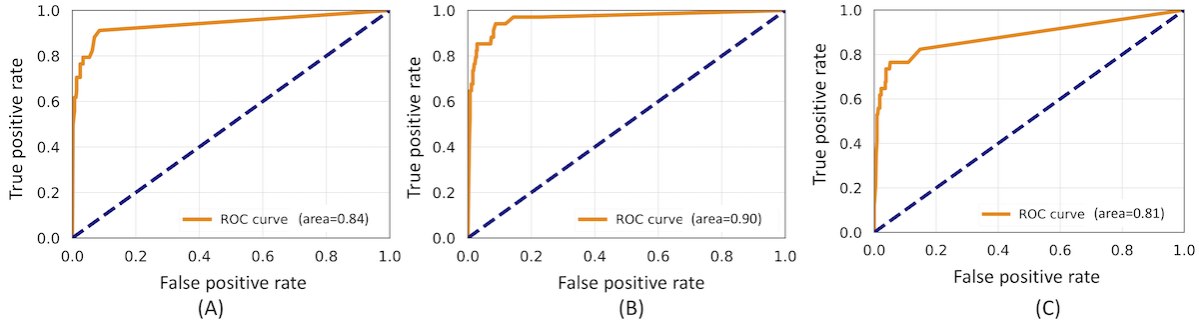
Receiver operating characteristic (ROC) curves for each classifier. Training and test data drawn from 2400 opioid-related posts from Twitter, web-based health communities, and YouTube. (A) logistic regression+semantic; (B) logistic regression+term frequency-inverse document frequency; and (C) long short-term memory+bidirectional encoder representations from transformers.

Table 1 and Figure 1 show the best-performing ML models in terms of their area under the curve, precision, recall, and F_1_ scores. Our best-performing model was a combination of TF-IDF features and an LR classifier, achieving a precision of 0.85, a recall of 0.91, an F_1_ score of 0.88, and an area under the curve of 0.9. By applying our best-performing model to machine label all posts in our data sets, we were able to estimate the prevalence of posts related to the myth under investigation on each platform. The prevalence of posts among our sampled comments that were related to the myth that the use of MOUD does not constitute true recovery was 0.4%, 0.9%, and 0.58% for web-based health communities, Twitter, and YouTube, respectively. For additional context and interpretability in terms of how our best-performing models operated per platform, 2 examples of posts that were classified correctly by our classifier are provided in Table 2, along with the top words used by the classifier to attain a relevancy decision for each post on each platform. Here we observed some consistencies in the discussions of the myth across platforms. For example, we noted that our model was able to pick up on the use of verbs synonymous with *replac*, such as *switch*, which was not originally included in the myth phrasing. In addition, the verb *go* was used in multiple contexts, such as going to Alcoholics Anonymous meetings instead of relying on MATs and going through withdrawals from MAT. We also noted the presence of multiple drug names such as *Ativan*, *buprenorphine*, *methadone*, and *suboxone*.

**Table 1:** Macroperformance metrics of the opioid use disorder treatment myth classifiers^a^.

Model	Accuracy	AUC^b^	Precision	Recall	F_1_ score
LR^c^+semantic^d^	0.84	0.84	0.87	0.86	0.86
LR+TF-IDF^e^	0.91	0.9	0.85	0.91	0.88
LSTM^f^+BERT^g^	0.77	0.81	0.83	0.84	0.84

^a^Training and test data drawn from 2400 opioid-related posts from Twitter, web-based health communities, and YouTube.

^b^AUC: area under the curve.

^c^LR: logistic regression.

^d^InferSent semantic representations (4096 features).

^e^TF-IDF: term frequency-inverse document frequency.

^f^LSTM: long short-term memory.

^g^BERT: bidirectional encoder representations from transformer.

**Table 2:** Paraphrased examples detected by our best-performing classifier on different platforms and top features highlighted. Raw posts are paraphrased to prevent traceability and author identification.

Platform and raw paraphrased post			Preprocessed post		Feature power		
					Contribution		Feature
**Web-based health communities**							
	“Don’t take the kratom. Don’t switch one drug for another. Go to an aa meeting. for real. IV Ativan is usually the go to drug for such symptoms.”	“take kratom switch one drug anoth go aa meeting for real iv ativan usual go drug symptom”		+2.955 +2.055 +1.783 +1.710 +1.585 +1.479 +1.251 +1.238 +1.057		ativan go usual drug aa anoth one symptom switch	
	“[...] [Name of a person] said: Please dont take it! If you can stop using opiates and not go back just go through the withdrawals. If you would trust me, you dont want the withdrawals (especially long term) that Bupe has! Please know that the length of the withdrawal period for maintenance users is in part dependent on the dose [...]”	“[...] [Name of a person] said pleas take stop use opiat go back go withdraw promis want withdraw especi long term bupe pleas understand length withdraw period mainten user part dose depend [...]”		+1.080 +0.734 +0.693 +0.691 +0.559 +0.510 +0.475 +0.460 +0.435 +0.427		therapi buprenorphin dose mainten replac go need appropri pain may	
**Twitter**							
	“Saying that people dying of Heroin/Fentanyl ODs^a^ is because they are getting Rx meds from doctor is just irresponsible & untrue. When someone gets addicted to methadone, what is happening is that $$ from the street are getting switched to $$ to big Pharma & our GOV. Abusing methadone/Suboxone still leads to deaths.”	“say rx med dr mani die heroin fentanyl od simpli irrespons untru get someon addict methadon simpli switch street go big pharma gov peopl still die abus methadon suboxon”		+2.41 +1.750 +1.142 +0.814 +0.752 +0.732 +0.589 +0.398 +0.376		methadon simpli med suboxon switch go irrespons mani get big	
	“I wonder if the w/d from Bupe or Suboxone is any easier than heroin or fentanyl. Let’s say someone switched to MAT^b^ as an interim because they wanted to be substance free; do you think they would go through w/d 2x?”	“w bupe suboxon easier heroin fentanyl one want substanc free would one go w x one switch mat interim”		+4.095 +3.801 +1.418 +1.157 +1.071 +1.041 +0.922 +0.583 +0.188		mat one bupe suboxon switch go easier substanc heroin want	
**YouTube**							
	“Okay I am planning to discontinue treatment. I feel I need support, but with my family disapproving of this treatment of being on MMT^c^, I don’t seem to be getting that. To them, it is no different from doing heroin everyday. They say I am switching one addiction for another [...]”	“decid discontinu treatment famili agre form treatment im get support mmt dont see differ heroin everyday say switch one addict anoth [...]”		+3.722 +3.595 +1.857 +1.290 +1.076 +0.577 +0.393 +0.385 +0.332		methadon treatment anoth go suboxon form one mmt get switch	
	“Your fear of the withdrawal symptoms is totally legit. They suck. Did you tell your doctor about your intake of the prescription? There needs to be some sort of a planned approach for not just quitting, but also to make sure you ween off your meds properly. Have you heard of Suboxone? It’s a prescription medication that basically will help you with withdrawals as well as give you a crutch. Kratom is another option, but going through the withdrawal alone and learning how to walk away as a substance-free person takes a lot of daring and audacity, so you need to have what it takes for it.”	“fulli understand fear withdraw symptom suck doctor know intak prescript game plan set quit also effort ween med sure heard suboxon prescript medic short summari itll help withdraw well act like crutch anoth thing kratom go withdraw one one learn walk away medfre person take lot gut courag take”		+1.370 +1.009 +0.878 +0.810 +0.780 +0.704 +0.678 +0.658 +0.626 +0.606		one prescript med anoth medic quit symptom effort suboxon set	

^a^OD: overdose.

^b^MAT: medication-assisted treatment.

^c^MMT: methadone maintenance treatment.

The top 10 features (terms) associated with our best-performing model (LR+TF-IDF) for identifying relevant posts and their TF-IDF values are shown in Table 3. These terms include *mat*, *assist*, *treatment*, *replac*, *therapi*, *rehab*, *methadon*, *behavior*, *habit*, and *substitut*. Furthermore, to provide additional insight into words used by the ML model to identify myth-related posts, for each of the top 10 terms, we display the 15 words with the closest semantic proximity (based on training a Word2Vec embedding model [48]) as measured by cosine similarity. Qualitative assessment of the identified words revealed excellent identification of synonymous terms and phrases, including those that were unlikely to be readily suggested or identified by human readers, such as *ost* (opioid substitution therapy).

**Table 3:** Top 10 salient features and their associated Word2Vec model nearest neighbors per platform^a^.

Feature and platform		Nearest neighbors
**mat (14.9)**		
	Web-based health communities	assist (0.49), proven (0.46), lifer (0.46), abstin (0.42), recoveri (0.41), stigma (0.41), mmt (0.41), superior (0.4), vivitrol (0.39), align (0.39), treatment (0.39), lifesav (0.39), mainten (0.39), adhes (0.39), bamboo (0.38)
	Twitter	treatment (0.61), medic (0.48), suboxon (0.46), bupe (0.43), need (0.4), therapi (0.39), behavior (0.39), stigmat (0.38), oud (0.38), postod (0.38), clear (0.37), suffici (0.37), med (0.36), part (0.36)
	YouTube	assist (0.72), recommend (0.72), care (0.7), bullshit (0.68), recoveri (0.67), truli (0.66), mention (0.66), step (0.65), anyon (0.65), mani (0.64), could (0.64), oud (0.63), possibl (0.63), lose (0.62), integr (0.62)
**assist (12.44)**		
	Web-based health communities	mat (0.49), counsel (0.45), profession (0.44), supervis (0.42), lifesav (0.4), help (0.38), certifi (0.38), vivitrol (0.38), aftercar (0.37), florida (0.37), longterm (0.37), mainten (0.37), recoveri (0.36), consult (0.36), transit (0.36)
	Twitter	appropri (0.4), profession (0.37), switzerland (0.36), mat (0.36), aaap (0.35), grade (0.34), staff (0.34), necessary (0.33), ongo (0.33), treatment (0.33), discrimin (0.32), center (0.31), evidenc (0.31)
	YouTube	famili (0.79), medic (0.77), judg (0.73), mat (0.72), recommend (0.71), lose (0.71), mani (0.71), could (0.7), therapi (0.7), battl (0.69), wonder (0.69), truli (0.67), win (0.67), recoveri (0.65), group (0.63)
**treatment (11.43)**		
	Web-based health communities	program (0.52), evid (0.51), ibogain (0.51), nation (0.51), medic (0.5), assess (0.49), longterm (0.48), wherein (0.48), addict (0.48), establish (0.47), intervent (0.46), protocol (0.46), rehabilit (0.46), observ (0.46), augment (0.46)
	Twitter	medic (0.67), therapi (0.66), mat (0.61), use (0.6), postod (0.59), need (0.55), drug (0.53), opioid (0.52), methadone (0.52), patient (0.51), reduc (0.48), rehab (0.48), provid (0.46), prescrib (0.45)
	YouTube	individu (0.72), treat (0.65), truli (0.65), ibogain (0.64), acknowledg (0.64), oud (0.62), recoveri (0.62), comfort (0.62), assist (0.61), receiv (0.6), great (0.6), keep (0.59), wonder (0.59), bullshit (0.56), worri (0.55)
**replac (9.91)**		
	Web-based health communities	swap (0.44), substitut (0.41), exercis (0.39), switch (0.39), fix (0.38), hormon (0.38), lifestyl (0.37), atom (0.37), still (0.36), healthi (0.35), discomfort (0.35), slowli (0.34), bad (0.34), lead (0.33), use (0.33)
	Twitter	substitut (0.48), altern (0.42), simpli (0.37), adjunct (0.35), extrem (0.35), swap (0.35), scienc (0.34), type (0.34), neither (0.32), panacea (0.32), creat (0.32), reduc (0.32), result (0.32), lifelong (0.31), grade (0.3)
	YouTube	hip (0.74), due (0.58), lot (0.57), altern (0.55), complet (0.55), result (0.54), k (0.54), rapid (0.53), someth (0.51), realiti (0.49), exchang (0.49), would (0.48), anti (0.47), argu (0.47), told (0.47)
**therapi (9.43)**		
	Web-based health communities	counsel (0.61), cbt (0.57), trauma (0.54), dbt (0.54), somat (0.49), therapist (0.48), ptsd (0.47), aftercar (0.46), tool (0.46), cognit (0.46), treatment (0.46), adjunct (0.45), psychiatri (0.45), longterm (0.45)
	Twitter	treatment (0.66), medic (0.44), psycholog (0.43), sizabl (0.43), psychosoci (0.43), acupunctur (0.43), use (0.42), postod (0.41), howev (0.41), incl (0.4), mat (0.39), success (0.37), pain (0.37), odb (0.37), need (0.37)
	YouTube	group (0.81), recoveri (0.76), na (0.74), requir (0.71), assist (0.7), oud (0.69), famili (0.69), recommend (0.67), aa (0.66), set (0.66), individu (0.64), base (0.64), great (0.63), bullshit (0.59), mat (0.59)
**rehab (8.45)**		
	Web-based health communities	inpati (0.59), facil (0.55), detox (0.55), centr (0.51), outpati (0.51), relaps (0.49), iop (0.49), ua (0.49), sober (0.48), homeless (0.47), residenti (0.47), jail (0.46), program (0.46), na (0.46), voluntarili (0.44)
	Twitter	treatment (0.48), residenti (0.46), mandatori (0.43), get (0.39), staffer (0.38), drug (0.38), go (0.37), one (0.37), clean (0.35), sobrieti (0.34), need (0.33), whitewash (0.33), mostli (0.33), let (0.33)
	YouTube	went (0.83), show (0.77), gone (0.76), new (0.7), bottom (0.67), bare (0.67), littl (0.63), day (0.62), gonna (0.62), sadli (0.6), away (0.6), process (0.59), gave (0.59), mom (0.54), keep (0.53)
**methadon (8.43)**		
	Web-based health communities	suboxon (0.78), heroin (0.57), opiat (0.57), bupe (0.52), oxi (0.5), clinic (0.5), sub (0.49), taper (0.49), mainten (0.48), mmt (0.48), dope (0.45), stigma (0.45), detox (0.45), addict (0.45)
	Twitter	treatment (0.52), opioid (0.46), drug (0.46), medic (0.42), use (0.41), postod (0.39), base (0.38), residenti (0.38), option (0.37), continu (0.37), provid (0.37), mani (0.36), client (0.36)
	YouTube	trust (0.68), switch (0.66), without (0.65), scare (0.62), suboxon (0.61), im (0.6), hate (0.59), due (0.59), anyway (0.56), year (0.56), dose (0.53), transit (0.53), wait (0.51), yr (0.51), center (0.51)
**behavior (8.14)**		
	Web-based health communities	behaviour (0.52), empathi (0.49), eif (0.44), repetit (0.44), undetect (0.44), destruct (0.44), hostil (0.43), cbt (0.43), exhibit (0.43), pattern (0.42), drugseek (0.42), flexibl (0.42), manipul (0.42)
	Twitter	physic (0.39), behaviour (0.39), mat (0.38), topamax (0.37), workflow (0.36), yoga (0.36), cognit (0.35), nprzyb (0.35), multilevel (0.35), recogn (0.35), rank (0.34), diseas (0.33), group (0.33), kneepain (0.33), approach (0.33)
	YouTube	jail (0.9), interest (0.89), servic (0.87), grant (0.87), integr (0.84), organ (0.83), learn (0.8), via (0.79), find (0.77), healthcar (0.77), health (0.77), final (0.75), set (0.74), mani (0.72), educ (0.71)
**habit (7.96)**		
	Web-based health communities	struggl (0.52), willpow (0.5), allen (0.48), carr (0.48), smoke (0.48), stop (0.45), habit (0.45), cig (0.45), cigarette (0.44), feel (0.42), go (0.42), definit (0.41), sobrieti (0.4), time (0.4), smoker (0.4)
	Twitter	crack (0.39), googlawaqpp (0.37), dailyrecord (0.36), pushi (0.36), rehab (0.33), bright (0.33), intox (0.33), black-watch (0.32), mccain (0.32), filthi (0.32), iff (0.31), weed (0.31), sober (0.31)
	YouTube	herion (0.74), beer (0.58), slave (0.54), codein (0.54), trade (0.53), chemic (0.52), far (0.52), issu (0.52), kratom (0.51), compound (0.5), anoth (0.49), wake (0.49), immedi (0.49), sick (0.48), evil (0.48)
**substitut (7.65)**		
	Web-based health communities	deriv (0.47), replac (0.45), sert (0.45), synthes (0.44), indol (0.44), halogen (0.43), amin (0.43), keton (0.41), phenyl (0.41), monocycl (0.41), hydrogen (0.4), piperidin (0.4), haloalkyl (0.39)
	Twitter	replac (0.47), ost (0.35), psilocybin (0.33), dcr (0.31), lesser (0.31), hepatitisc (0.3), licat (0.3), abstain (0.29), deaden (0.29), halflif (0.28), assist (0.28), cab (0.28)
	YouTube	anoth (0.69), sell (0.69), address (0.67), none (0.67), slave (0.65), exchang (0.63), isnt (0.61), what (0.61), crutch (0.6), issu (0.59), sinc (0.58), there (0.58), trade (0.57), meant (0.55), unbroken (0.54)

^a^Data from 112,281 opioid-related posts identified by our best-performing model from Twitter, web-based health communities, and YouTube. The first column depicts the features and their term frequency-inverse document frequency scores. The nearest neighbors column also depicts the cosine similarity between each word and the corresponding feature. Words in posts are stemmed before being fed to models (eg, recovery is stemmed to its root *recoveri*). Web-based health communities refer to Reddit and Drugs-Forum.

## Discussion

### Principal Findings

Harms propagated by misinformation are aplenty on the web and come at both financial and societal costs. People often accept what they read as true, especially if it comes from a reasonably reputable source, and do not question the information, no matter how astounding or alarming. In fact, people even repeat the more remarkable information regardless of how accurate it is. In the context of MOUD, it can lead to grave consequences, including overdose deaths [29]. To the best of our knowledge, this is the first study to examine MOUD-related misinformation on a large scale, harnessing conversations happening on the web.

Closely related to our work is the study by Jamison et al [49], which leverages a collection of tweets to quantify vaccine misinformation. Similar to our work, Jamison et al [49] coded tweets into thematic categories based on vaccine sentiment (positive, negative, or neutral). However, our work leveraged thematic categories (relevant and not relevant to the myth) to design ML-based models that are able to identify misinformation in the context of MOUDs. Heimer et al [29] discussed prevalent misconceptions about OUDs in the United States through 3 crises (1865-1913, 1960-1975, and 1995-today). Similar to our focus, the authors acknowledged opioid *abstinence-based* recovery models as a prevailing misconception and promoted the large-scale expansion of MAT. Our work complements their work by investigating this misconception quantitatively through the lens of social media. Chenworth et al [50] investigated the perception of the general public toward methadone and buprenorphine-naloxone on Twitter. The authors identified that a common barrier to treatment with these medications was the idea of opioid substitution—the exchange of one opioid addiction for another [50]. Our work investigates this barrier at a deeper level by building models that are able to recognize this type of discourse on social media.

Our results have important public health implications. Across multiple platforms, we detected that the prevalence of posts about a single myth related to medication treatment for OUD in our sample ranged from 4 per 1000 posts on web-based health communities to 9 per 1000 posts on Twitter. This is notable, as, at any time, there are likely multiple myths being discussed on the web, suggesting that the total volume of misinformation content related to opioids may be a substantial proportion of the total posts. The prevalence of such information has not been previously quantified, and this study offers important insights into the potential scope of this health information issue.

Although we cannot speculate on the exact reason why Twitter presented more misinformation in the case of OUD-related misinformation as that requires causal inference analysis, which is beyond the scope of this paper, prior literature has pointed out the lack of active expert or clinical-based moderation on Twitter [51]. Although web-based health communities are also not immune to bad behavior and antisocial activities such as trolling, spamming, and harassment, these communities are often guided by strict norms against such behavior and moderated to ensure the quality and credibility of the content being shared [52]. Prior studies on different types of web-based health communities have demonstrated that adequate active moderation increases the engagement of members and consequently also increases the beneficial outcomes for members in a web-based community [53]. In fact, the moderators themselves regard their moderation style as important for the regulation and stimulation of membership engagement [54,55]. We suspect that, because of these established moderation norms, we observed a relatively lesser prevalence of MOUD misinformation in the web-based communities we studied. We noted that Twitter does implement some broad governance rules that allow for certain types of information to stay on the platform, whereas others are removed (eg, graphic violence and adult content [56]). The platform also has provisions to tackle the widespread presence of hate speech and abusive content [57]. However, to the best of our knowledge, Twitter does not implement policies toward the moderation of MOUD misinformation. Our conjecture is that, because of this existing practice, our study revealed a greater prevalence of this misinformation on the platform. Nevertheless, in light of the ongoing COVID-19 pandemic, Twitter has broadened its definition of harm to address “content that goes directly against guidance from authoritative sources of global and local public health information” [58]. We hope that the findings of this study can motivate social media platforms to consider moderation approaches toward substance misuse information as well.

Given the significant prevalence of myths around OUD treatment, as shown in this study, a possible approach to counter web-based misinformation could be to perform targeted, expert fact-checking of social media posts. This could mirror and harness guidelines adopted by public health organizations to debunk unverified information about OUD treatment. For instance, substance use experts can be identified and asked to review the content of social media posts to determine their accuracy. These experts could critically appraise a post and produce a response comprising a lay summary of the evidence in addition to a detailed, referenced evidence review. This review could be directly linked to the original post through appropriate platform affordances to provide users with quick access to fact-checked information. Specific fact-checking processes could also be tailored to individual social media platforms, given the differences we observed both in terms of prevalence and the linguistic characteristics of the myth discussions. Qualitative exploration of the characteristics of the statements identified by the ML approach revealed linguistic and topical diversity. Some statements explicitly referenced the main concept we queried for—that MOUD represents replacing one drug with another. However, related statements were identified in which alternative treatments such as kratom entered into the discussion. Rationales for hesitancy toward MOUD also became apparent, including concerns about the addictiveness of MOUD, the nature of withdrawal symptoms from MOUD, and concerns about industry or governmental motivations for recommending MOUD. Understanding these concerns is directly relevant to providing health information, understanding the role of digital information ecosystems as a supplant or adjuvant resource in substance misuse treatment, and addressing treatment hesitancy.

In addition to fact-checking efforts, public health engagement campaigns could also be used to address specific cases of misinformation. Recent research suggests that information campaigns led by trusted community members and health partners can help address health misinformation on social platforms [59]. Accordingly, alliances can be forged with social media influencers and key opinion leaders to run targeted health promotion campaigns. Interventions such as those with positive messaging can also be tailored to the preferences, perceptions, and cultures of different platforms. Educational interventions that improve literacy around OUD treatment and reduce the stigma that precludes seeking help, as well as ecologically sensitive interventions that open up avenues to access social support, could also empower individuals to be better equipped to deal with OUD treatment myths on the web. In short, although the literature on strategies to effectively counter health misinformation is still emerging, at minimum, this work highlights the importance of ongoing assessment and awareness of what health information is being prominently discussed on the web to guide both the provision of effective health care and public health prevention activities.

We note some limitations of this work. Although our analysis included large data sets from diverse web-based platforms, MOUD-related discussions happen on a wide variety of social platforms, and the prevalence of misinformation across a broader set of web-based environments needs characterization. For one platform, YouTube, limitations in the number of comments that can be accessed required restriction of the keyword list, which may have affected the prevalence of misinformation, although the estimate from YouTube was comparable with the other platforms. Furthermore, this research did not examine the nature of conversations surrounding the OUD treatment myth we focused on in this paper, such as whether a conversation might be reinforcing or countering the myth or discussing other previously known myths. Future work may unpack these characteristics of web-based discussions while also investigating additional myths about OUD misuse that surface on web-based platforms. Finally, geospatial-temporal studies on MOUD misinformation that originates and spreads via social media platforms can be a promising and significant direction for future research; they can influence interventions such as targeted location-based misinformation-countering campaigns as well as help clinicians respond to patients’ false beliefs or misperceptions.

### Conclusions

Using ML and natural language analysis, our research demonstrated promise in identifying social media posts that centered on treatment myths about OUD, including how these discussions varied across platforms in terms of prevalence. As the overdose epidemic continues to evolve, attention from health professionals to health information on the web that drives patient decision-making will continue to be a critical element of prevention.

## Abbreviations

CDC: Centers for Disease Control and Prevention
KNN: k-nearest neighbor
LR: logistic regression
LSTM: long short-term memory
MAT: medication-assisted treatment
ML: machine learning
MOUD: medication for opioid use disorder
OUD: opioid use disorder
TF-IDF: term frequency-inverse document frequency
